# Mycosis fungoides in the setting of 9p minus syndrome

**DOI:** 10.1016/j.jdcr.2026.07.051

**Published:** 2026-08-01

**Authors:** Joseph Lozenski, Peter Chow, Sarah Pourakbar, Jordan A. Dykstra

**Affiliations:** a University of Kansas School of Medicine, Kansas City, Kansas; b University of Kansas Medical Center, Division of Dermatology, Kansas City, Kansas

**Keywords:** 9p minus (9p-) syndrome, CTCL, mycosis fungoides, oncodermatology, oncogenes

## Background

Mycosis fungoides (MF) is a variant of cutaneous T-cell lymphoma (CTCL) that originates in peripheral epidermotropic T-cells. MF accounts for approximately 60% of CTCL diagnoses and predominantly affects adults over the age of 50. MF lesions may progress to involve the lymph nodes and spread hematogenously.^1^ Chromosomal analyses of the atypical lymphocytes in patients with MF have demonstrated allelic loss of chromosome 9p.^2^ Complete loss of 9p may promote clonal proliferation, as 9p21 encodes the tumor suppressor genes CDKN2A and CDKN2B. CDKN2A and CDKN2B encode proteins P15 and P16, which play critical roles in cell-cycle regulation.^2^ Genetic loss of CDKN2A-CDKN2B was significantly associated with shorter survival time of CTCL patients.^3^ We report a novel case of MF occurring in the setting of underlying 9p minus (9p-) syndrome. 9p- syndrome is a rare autosomal dominant disorder that is due to allelic loss of the short arm of chromosome 9. It presents with clinical features of craniofacial dysmorphism and hypertonia. 9p- syndrome may also present with neurodevelopmental disorders, behavioral disorders and seizure disorders.^4^^,^^5^

## Case presentation

**Fig 1:**
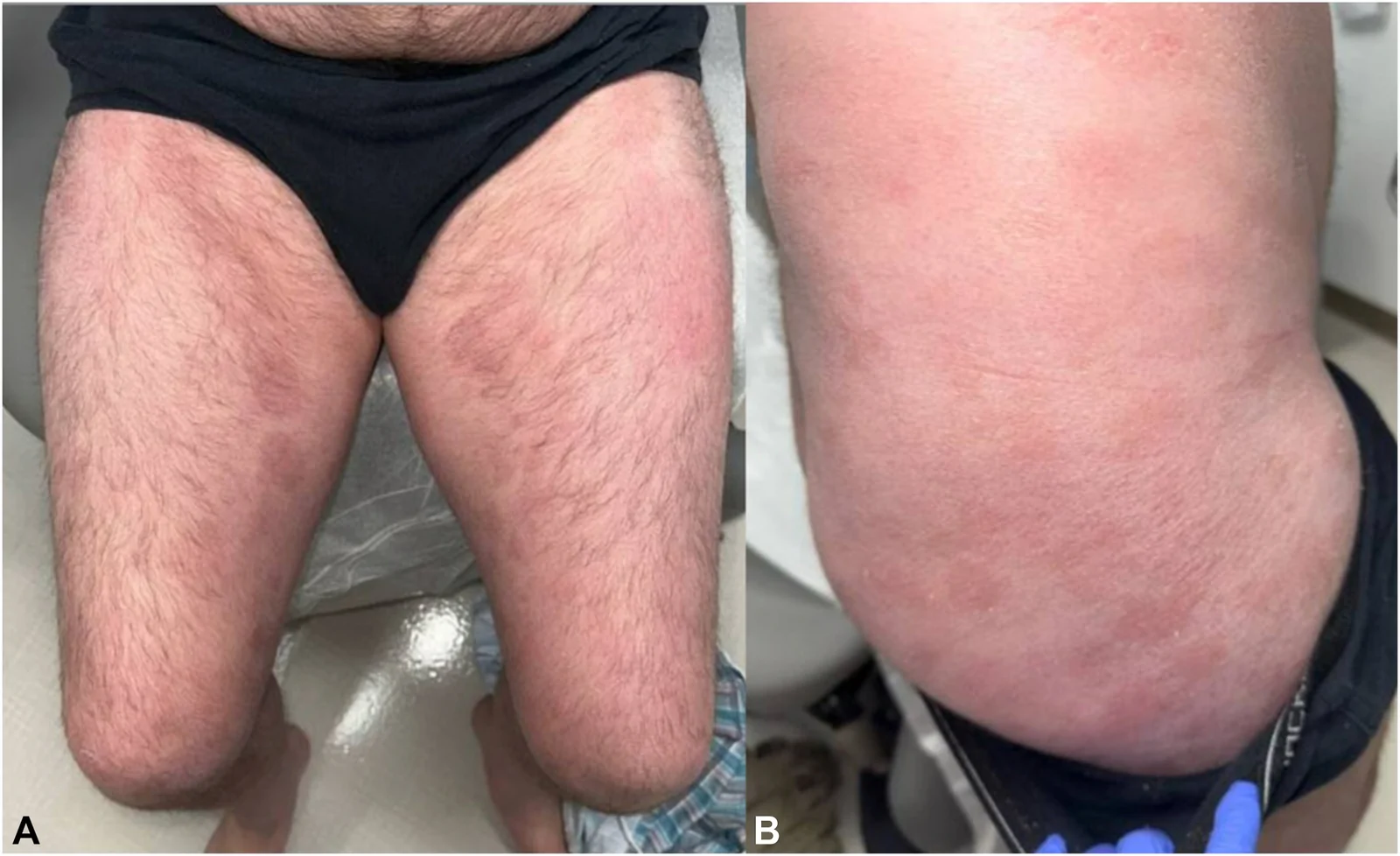
**A,** Clinical photograph of active MF lesions involving bilateral thighs. **B,** Clinical photograph of active MF lesions involving right buttock and flank. Images obtained at initial dermatology clinic presentation.

**Fig 2:**
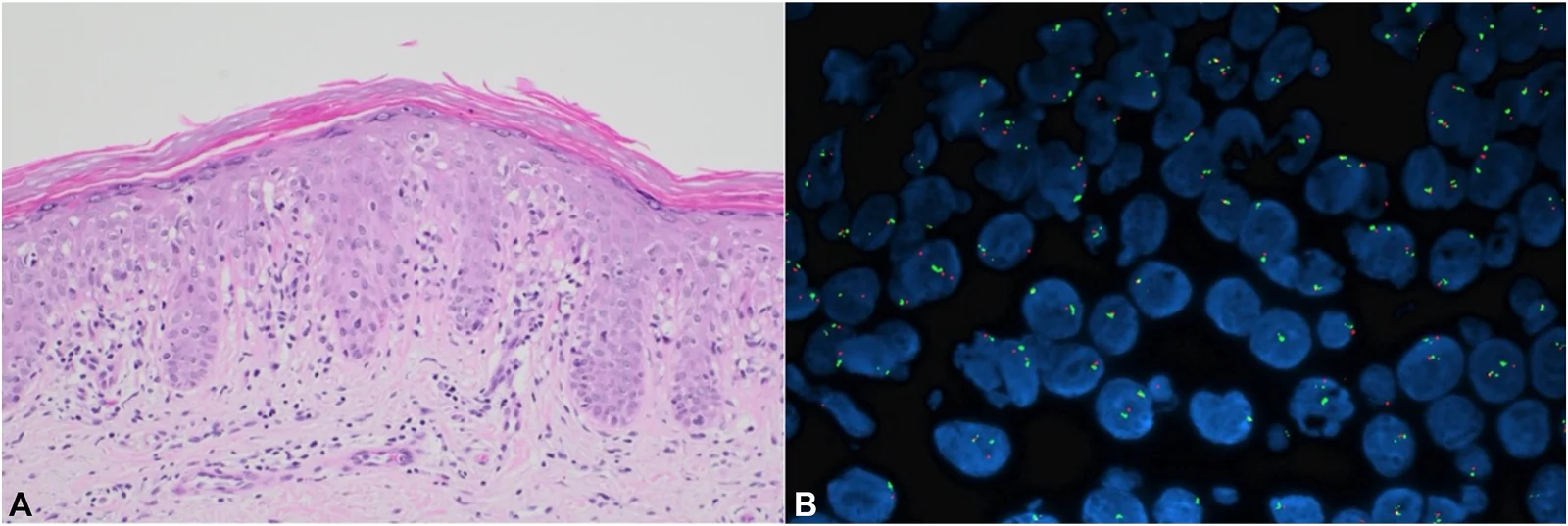
**A,** Histopathologic examination of the right buttock biopsy specimen demonstrating atypical lymphocytic infiltrate on hematoxylin and eosin staining (original magnification x20). **B,** FISH analysis of formalin-fixed right buttock biopsy tissue demonstrating CDKN2A deletion.

A 31-year-old male presented to the dermatology department for a mildly pruritic eruption that had started a year prior and persisted despite treatment with topical emollients and antifungals. The eruption initially involved the inguinal and gluteal regions and later progressed to diffuse xerosis sparing the head with multiple pink, scaly, ovoid plaques predominantly in sun protected areas (Fig 1). At presentation, total body surface area involved was greater than 50%. There was no lymphadenopathy or organomegaly. The patient had a past medical history consisting of 9p- Syndrome, autism spectrum disorder, generalized epilepsy, attention-deficit/hyperactivity disorder, type 2 diabetes mellitus, and hypothyroidism. The initial differential diagnosis included a high suspicion for mycosis fungoides, given the distribution, clinical appearance, and history. Other papulosquamous dermatoses were included on the differential but were considered less likely. Broad shave biopsies obtained from the right buttock and thigh both demonstrated abundant small to medium sized T cells highlighted by CD3 in the epidermis. The CD4:CD8 ratio was mildly skewed at approximately 8:1. There was partial CD7 loss in the epidermotropic lymphocytes. CD30 was negative. There was no significant loss of expression of CD5. A clonal T-cell gene rearrangement was detected in the right buttock tissue sample (Fig 2, *A*). Further laboratory analysis revealed an LDH of 201, a negative Sezary cell count, and normal peripheral blood flow cytometry results. A FISH analysis revealed a loss of CDKN2A (9p) in 45% of nuclei (Fig 2, *B*). These cells also demonstrated deletion of the chromosome 9 centromere, consistent with monosomy 9.

The patient was diagnosed with stage Ib mycosis fungoides and started on narrow band UV-B phototherapy, topical hydrocortisone 2.5% ointment to intertriginous sites, and topical triamcinolone 0.1% ointment to trunk and extremities. At the 3 month follow up visit, patient reported significant improvement in cutaneous symptoms with total body surface area involved less than 2%. At the 6 month follow up, no active MF lesions were observed.

## Discussion

To our knowledge, this is the first reported case of MF in a patient living with 9p- syndrome. The patient’s FISH results indicate that the clonal T-cells share the same chromosomal loss as the patient’s germline mutation. Based on prior studies, this finding may suggest that the patient may harbor a more aggressive variant of MF with shortened overall survival.^3^ This may prompt increased frequency of follow-ups, systemic monitoring, and may impact treatment decisions in the future. Currently, however, the patient is doing well on skin directed therapy. While 9p- syndrome is rare, our observation raises the possibility that patients living with 9p- syndrome may be predisposed to developing MF. Further studies should be completed to examine a relationship between 9p- syndrome and the development of MF. If MF develops, the next step should be closely following these patients to monitor the aggressiveness of the disease course.

### Declaration of generative AI and AI-assisted technologies in the writing process

During the preparation of this case report, the authors did not use any artificial intelligence tool or service.

## Conflicts of interest

None disclosed.
